# Diversity and interactions of rhizobacteria determine multinutrient traits in tomato host plants under nitrogen and water disturbances

**DOI:** 10.1093/hr/uhae290

**Published:** 2024-10-15

**Authors:** Wen-Xuan Shi, Jun-Jie Guo, Xin-Xuan Yu, Zhi-Xing Li, Bo-Yang Weng, Dan-Xia Wang, Shi-Hao Su, Yu-Fei Sun, Jin-Fang Tan, Ruo-Han Xie

**Affiliations:** State Key Laboratory of Biocontrol, Guangdong Provincial Key Laboratory of Plant Stress Biology, School of Agriculture and Biotechnology, Shenzhen Campus of Sun Yat-sen University, Sun Yat-sen University, Shenzhen, Guangdong 518107, China; State Key Laboratory of Biocontrol, Guangdong Provincial Key Laboratory of Plant Stress Biology, School of Agriculture and Biotechnology, Shenzhen Campus of Sun Yat-sen University, Sun Yat-sen University, Shenzhen, Guangdong 518107, China; State Key Laboratory of Biocontrol, Guangdong Provincial Key Laboratory of Plant Stress Biology, School of Agriculture and Biotechnology, Shenzhen Campus of Sun Yat-sen University, Sun Yat-sen University, Shenzhen, Guangdong 518107, China; State Key Laboratory of Biocontrol, Guangdong Provincial Key Laboratory of Plant Stress Biology, School of Agriculture and Biotechnology, Shenzhen Campus of Sun Yat-sen University, Sun Yat-sen University, Shenzhen, Guangdong 518107, China; State Key Laboratory of Biocontrol, Guangdong Provincial Key Laboratory of Plant Stress Biology, School of Agriculture and Biotechnology, Shenzhen Campus of Sun Yat-sen University, Sun Yat-sen University, Shenzhen, Guangdong 518107, China; State Key Laboratory of Biocontrol, Guangdong Provincial Key Laboratory of Plant Stress Biology, School of Agriculture and Biotechnology, Shenzhen Campus of Sun Yat-sen University, Sun Yat-sen University, Shenzhen, Guangdong 518107, China; State Key Laboratory of Biocontrol, Guangdong Provincial Key Laboratory of Plant Stress Biology, School of Agriculture and Biotechnology, Shenzhen Campus of Sun Yat-sen University, Sun Yat-sen University, Shenzhen, Guangdong 518107, China; State Key Laboratory of Biocontrol, Guangdong Provincial Key Laboratory of Plant Stress Biology, School of Agriculture and Biotechnology, Shenzhen Campus of Sun Yat-sen University, Sun Yat-sen University, Shenzhen, Guangdong 518107, China

## Abstract

Coevolution within the plant holobiont extends the capacity of host plants for nutrient acquisition and stress resistance. However, the role of the rhizospheric microbiota in maintaining multinutrient utilization (i.e. multinutrient traits) in the host remains to be elucidated. Multinutrient cycling index (MNC), analogous to the widely used multifunctionality index, provides a straightforward and interpretable measure of the multinutrient traits in host plants. Using tomato as a model plant, we characterized MNC (based on multiple aboveground nutrient contents) in host plants under different nitrogen and water supply regimes and explored the associations between rhizospheric bacterial community assemblages and host plant multinutrient profiles. Rhizosphere bacterial community diversity, quantitative abundance, predicted function, and key topological features of the co-occurrence network were more sensitive to water supply than to nitrogen supply. A core bacteriome comprising 61 genera, such as *Candidatus Koribacter* and *Streptomyces*, persisted across different habitats and served as a key predictor of host plant nutrient uptake. The MNC index increased with greater diversity and higher core taxon abundance in the rhizobacterial community, while decreasing with higher average degree and graph density of rhizobacterial co-occurrence network. Multinutrient absorption by host plants was primarily regulated by community diversity and rhizobacterial network complexity under the interaction of nitrogen and water. The high biodiversity and complex species interactions of the rhizospheric bacteriome play crucial roles in host plant performance. This study supports the development of rhizosphere microbiome engineering, facilitating effective manipulation of the microbiome for enhanced plant benefits, which supports sustainable agricultural practices and plant health.

## Introduction

Host plants, together with their associated microbiota, form a holobiont that exerts a significant influence on the regulation of plant health and productivity [1]. Plant holobiont research has predominantly focused on the assembly of microbial communities inhabiting the roots/rhizoplane/rhizosphere, as the soil–root interface represents a playground for plant–microbe coevolution [2–4]. The collective genome of microorganisms associated with the plant rhizosphere is enormous, much larger than that of the plant itself [5]. Thus, rhizosphere microorganisms have been recognized as the plant’s second genome, capable of forming functionally rich symbioses with plants and playing a crucial role in enhancing host nutritional status and overall health [6, 7]. However, our ability to harness the benefits of such interactions in the face of abiotic stress is severely hampered by a limited understanding of the microbiota roles that are integral to the holobiont.

Plant nutrient traits, particularly the elemental concentrations of potentially limiting nutrients in plant biomass (such as N, P, K, Ca, Fe, Mg, Zn, Cu, and Mn), exhibit significant correlations with plant phylogeny and ecosystem productivity [8]. Plant–microbe interactions can mold plant nutrient characteristics without altering the plant’s genomic information. For instance, the plant microbiota can control aspects of lateral root development via ethylene response induction in the root, thereby fostering plant growth and optimizing nutrient utilization [9]. Sole and combined additions of arbuscular mycorrhizal fungi and plant growth-promoting rhizobacteria enhanced root colonization, which led to increased N uptake in the root and shoot [10–12]. The synergies of arbuscular mycorrhizal fungi and saprotrophs facilitated plant nutrient uptake through enhanced enzyme activity [13]. Phosphorus-mobilizing rhizospheric microorganisms can lower the pH of the external environment, thereby regulating the available phosphorus in both soil and rock forms, which are inaccessible to plants [14]. However, most studies primarily address the role of rhizospheric bacteria in regulating single nutrient [15], and little is known about the pathways by which multiple nutrients are regulated. Rhizospheric bacterial communities drive multiple nutrient traits simultaneously instead of performing a single measurable process [16]. While the importance of rhizospheric bacterial communities in single-nutrient changes has been well established, their contributions to plant multinutrient traits remain unclear.

In recent years, industrial agricultural practices have led to the emergence of water and nutrient supply problems as potentially significant stressors for plant growth [17]. Nitrogen and water are important abiotic factors that determine the assembly of rhizospheric bacteria and play a crucial role in sustaining ecosystem multifunctionality [18]. The multinutrient cycling index (MNC), which quantitatively measures the levels of multiple nutrients by calculating the average of standardized scores for each individual nutrient [19], provides a promising alternative for simultaneously assessing the status of multiple plant/soil nutrients, similar to multifunctionality [20]. Current theories suggest the existence of a positive feedback loop between MNC and the assemblages of rhizospheric bacterial community [16]. The recruitment of specific active rhizospheric bacterial taxa appears to drive variation in plant multinutrient concentrations [16]. In turn, the multinutrient requirements of plants determine the rhizospheric bacterial assemblage [21]. Microbial diversity has been documented to enhance multinutrient cycling [22]. Multinutrient cycling is contingent upon not only the number of species within the community (microbial diversity), but also the intricate interrelationships among community members [23]. Moreover, the core microbiota plays a critical ecological role in the maintenance of complex associations among bacterial taxa and multinutrient cycling [19]. Despite the well-recognized relationship between microbial communities and multinutrient cycling, the relevance of rhizospheric bacterial community assemblages to MNC under the interaction of nitrogen and water is not well understood.

Tomato (*Solanum lycopersicum*) has a short growth cycle and is highly sensitive to nitrogen and water availability, thereby providing convenient phenotypic observation. Therefore, we used it as a model plant to characterize MNC in host plants subjected to different nitrogen and water supply regimes based on a greenhouse experiment. We employed absolute quantitative sequencing to investigate the impacts of nitrogen and water supply on microbial diversity and quantitative abundance, predict functions of rhizospheric bacterial communities, and explore the relative significance of their assemblages in host plant multinutrient cycles. Specifically, given the mechanistic links between bacterial communities and plant multinutrient traits, we hypothesized that multinutrient traits may be closely dependent on rhizospheric bacterial community assemblages, i.e. multinutrient absorption by host plants is primarily determined by the rhizobacterial community diversity and network complexity under the interaction of nitrogen and water. We expected this study to expand our ecological comprehension of the plant holobiont and provide new perspectives on the relationships between rhizospheric microbial community assemblages and plant functional traits. By revealing the assemblages of rhizosphere-inhabiting microorganisms, this work contributes a deeper understanding of the microbial and ecological mechanisms that govern plant multinutrient utilization under fluctuating environments.

## Results

### Differences in bacterial community diversity and quantitative abundance across treatments

**Figure 1:**
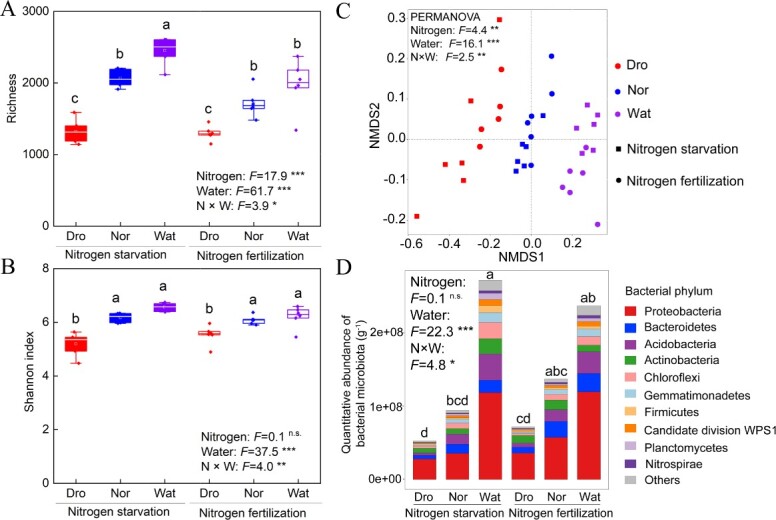
Diversity and quantitative abundance of microbial communities. (A, B) Richness and Shannon index of rhizospheric bacterial community in tomato. (C) NMDS plot based on the Bray–Curtis distance for rhizospheric bacterial community. Differences between treatments and their interactions were analyzed using PERMANOVA. Stress = 0.077. (D) Absolute abundance of major bacterial phyla in all treatments. Same letters indicate no significant difference at *P <* 0.05. *P < 0.05, **P < 0.01, ***P < 0.001. ns, not significant.

Water supply under nitrogen starvation and fertilization resulted in comparable alterations in bacterial community metrics (i.e. increases in bacterial richness and Shannon index) (Fig. 1a and b). The highest richness and Shannon diversity were observed in the waterlogged treatment, whereas the lowest values were noted for the drought treatment. Nitrogen and water interaction significantly affected richness (*F* = 3.9, *P* < 0.05) and Shannon diversity (*F* = 4.0, *P* < 0.01). Non-metric multidimensional scaling (NMDS) based on the Bray–Curtis distance combined with permutational multivariate analysis of variance (PERMANOVA) found that the bacterial microbiota formed three distinct clusters according to the water supply level (*F* = 16.1, *P* < 0.001), indicating a clear spatial ecological niche of the microbial communities (Fig. 1c**;**Supplementary Table S1). Quantitative assessment of bacterial cell abundance in the rhizosphere soil revealed that increased water supply led to an increase in quantitative abundance (*F* = 22.3, *P* < 0.001), and the interaction of nitrogen and water exhibited a notable effect on quantitative abundance (*F* = 4.8, *P* < 0.05) (Fig. 1d). Notably, the waterlogged treatment was associated with a significantly higher total bacterial load in the rhizosphere than the drought and normal water treatments.

### Predicted functional pathways

**Figure 2:**
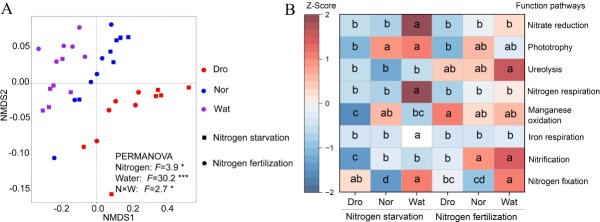
Predicted functional pathways of rhizospheric bacterial community. (A) NMDS plot for predicted functional pathways of rhizospheric community. Differences between treatments and their interactions were analyzed using PERMANOVA. Stress = 0.033. (B) Z-Scores of absolute abundance of predicted functional pathways in the tomato rhizosphere. Small letters indicate significant differences between six different treatments in one functional pathway. Same letters indicate no significant difference at *P <* 0.05. *P < 0.05, ***P < 0.001.

NMDS analysis revealed significant differences in the predicted functions of the bacterial community between nitrogen and water treatments (nitrogen: *F* = 3.9, *P* < 0.05; water: *F* = 30.2, *P* < 0.001) and their interaction (*F* = 2.7, *P* < 0.05) (Fig. 2a). In total, 43 predicted functional pathways were obtained using Functional Annotation of Prokaryotic Taxa (FAPROTAX), among which pathways associated with aerobic chemoheterotrophy, chemoheterotrophy, aerobic ammonia oxidation, nitrification, and nitrogen fixation were the most abundant (Supplementary Fig. S1). We focused on functional pathways associated with nutrient traits (Fig. 2b). We observed drastic alterations in the bacterial composition related to nitrogen fixation pathways under waterlogged conditions as compared with drought and normal water (*P* < 0.05). The quantitative abundance of functional pathways related to nitrogen metabolism was increased in waterlogged conditions at the same N level. Additionally, nitrogen fertilization resulted in a notable upregulation of the nitrification and ureolytic pathways when compared with nitrogen starvation under the same water supply level.

### Network topological feature responses to nitrogen and water supply

**Figure 3:**
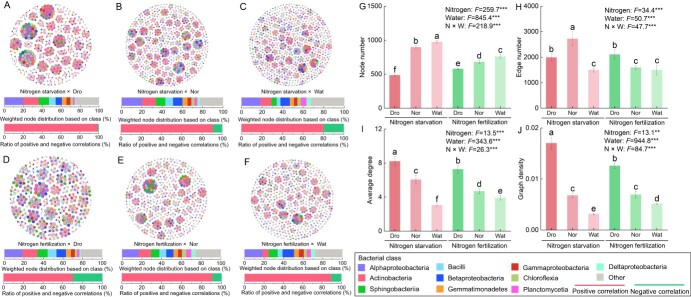
Co-occurrence patterns of rhizospheric bacterial communities as influenced by nitrogen and water supply. (A–F) Co-occurrence networks in soil bacteria under different treatments. Size of node proportional to number of connections. Only nodes with Spearman’s *ρ* > .7 (after Benjamini and Hochberg FDR adjustment, *P* < 0.05) were connected. (G–J) Number of nodes (G) and edges (H), average degree (I), and graph density (J) of networks under different treatments. Same letters indicate no significant difference at *P <* 0.05. **P < 0.01, ***P < 0.001.

The bacterial community showed distinct co-occurrence patterns under different treatments (Fig. 3). The classes *Alphaproteobacteria*, *Actinobacteria, Sphingobacteriia*, *Bacilli*, *Beyaproteobacteria*, and *Gemmatimonadetes* accounted for the majority of bacterial interactions (>50%) (Fig. 3a–f). The rate of positive associations was lower under drought plus nitrogen fertilization than under the other treatments (Fig. 3a–f). We used the number of nodes and edges, average degree, and graph density to represent the complexity of the microbial network, with higher values of these parameters indicating a greater level of network complexity. At the same nitrogen level, edge numbers, average degree, and graph density were higher under drought than under waterlogged conditions (*P* < 0.05), and these values decreased with increasing water supply (*P* < 0.001) (Fig. 3g–j). Node number (nitrogen: *F* = 259.7, *P* < 0.001; water: *F* = 845.4, *P* < 0.001), average degree (nitrogen: *F* = 13.5, *P* < 0.001; water: *F* = 343.6, *P* < 0.001), and graph density (nitrogen: *F* = 13.1, *P* < 0.01; water: *F* = 944.8, *P* < 0.001) were more sensitive to water supply than to nitrogen supply.

### Potential contribution of core taxon to plant nutrient traits and MNC

The concentrations of various nutrients in the aboveground shoot, including N, K, Fe, Mg, Zn, Cu, and Mn, were significantly affected by nitrogen, water, and their interaction (*P* < 0.05) (Supplementary Fig. S2). Under nitrogen starvation, nitrogen deficiency was alleviated under drought as compared with the normal water treatment. At the same nitrogen level, P and Mn concentrations increased with increasing water supply. Drought and waterlogged conditions enhanced Ca and Mg concentrations as compared to the normal water treatment. Under drought or waterlogged conditions, nitrogen fertilization significantly increased K, Fe, Zn, and Cu concentrations as compared with nitrogen starvation, whereas under the normal water treatment, there were no significant differences in nutrient concentrations between the nitrogen treatments (*P* > 0.05).

**Figure 4:**
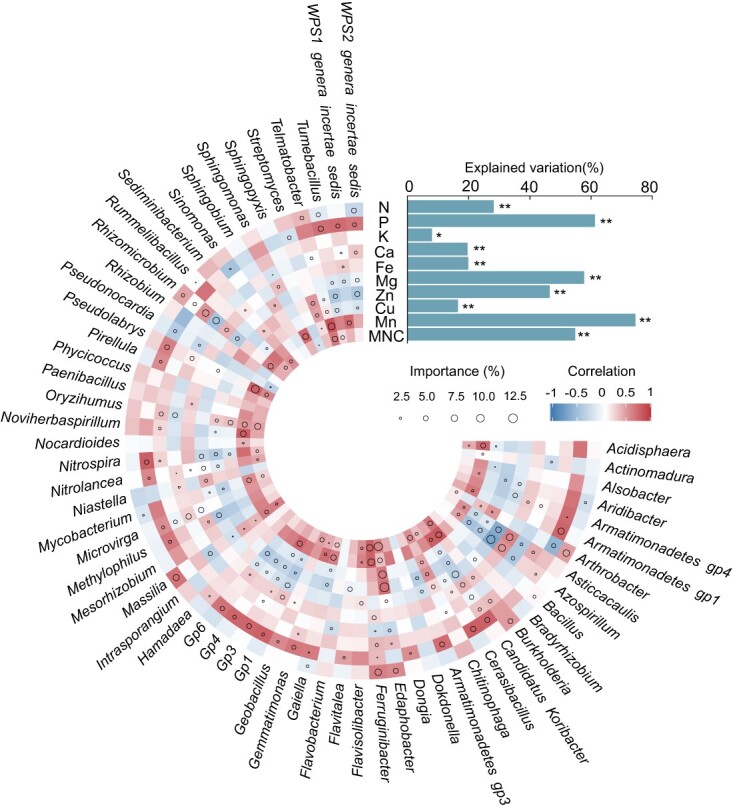
Potential biological contributions of genus-level core taxon on plant nutrient traits. Circle size represents the importance of a given variable. Colors represent Spearman correlations between nutrient concentrations and bacterial abundance. *P < 0.05, **P < 0.01.

We used random forest analysis to assess the biological associations of the quantitative abundances of core microbial taxa with variations in aboveground plant nutrient concentrations and MNC, which revealed that different bacterial abundances contributed to the changes in nutrient concentrations (Fig. 4). Specifically, the abundances of *Massilia*, *Ferruginibacter*, *Edaphobacter*, and *Burkholderia* were significant variables in predicting variations in plant N and Mn and showed a positive correlation with these nutrients. The abundances of *WPS1 genera incertae sedis*, *Pseudolabrys*, *Gp3*, *Gemmatimonas*, and *Candidatus Koribacter* were predictive of P and Mn concentrations. The abundances of *WPS2 genera incertae sedis*, *Tumebacillus*, *Nitrospira*, *Gp3*, *Gp1*, *Candidatus Koribacter*, *Azospirillum*, and *Asticcacaulis* were negatively correlated with Mg, Zn, and Cu concentrations. The abundances of *Microvirga*, *Candidatus Koribacter*, and *Asticcacaulis* were predictive of changes in K, Ca, and Mg concentrations. Moreover, the abundance of *Candidatus Koribacter* and *Nitrospira* was positively correlated with Fe concentration. The abundance of *Streptomyces* was positively correlated with Zn concentration. *Telmatobacter*, *Oryzihumus*, *Ferruginibacter*, *Edaphobacter*, *Chitinophaga*, and *Burkholderia* abundances were positively correlated with MNC, whereas *Edaphobacter* abundance was important in predicting changes in N, K, Zn, Cu, Mn, and the MNC index and was positively correlated with these parameters. Moreover, nitrogen fertilization increased MNC compared to nitrogen starvation, whereas drought decreased MNC when compared with the normal and waterlogged conditions (Supplementary Fig. S3).

### MNC and community diversity: network complexity, predicted functional abundance, and core taxon abundance

**Figure 5:**
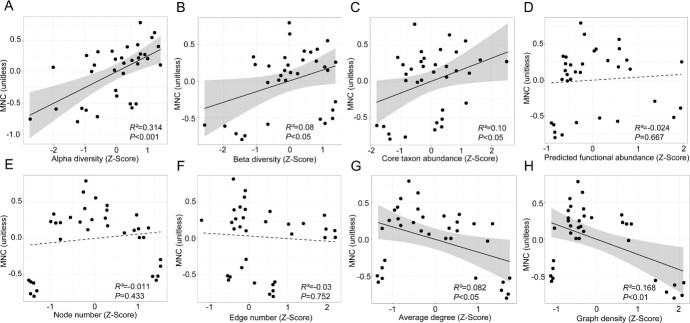
Relationships between MNC and community diversity, network complexity, predicted functional abundance, and core taxon abundance. Relationships between MNC and alpha diversity (A), beta diversity (B), core taxon abundance (C), predicted functional abundance (D), node number (E), edge number (F), average degree (G), and graph density (H). Gray-shaded regions depict 95% confidence intervals. Solid line indicates significant Pearson correlations (*P* < 0.05).

Significant positive relationships were observed between MNC and alpha diversity (*P* < 0.001), beta diversity (*P* < 0.05), and quantitative abundance of core taxon (*P* < 0.05), suggesting the pivotal role of the bacterial community in driving plant multinutrient traits (Fig. 5). Changes in network average degree (*P* < 0.05) and graph density (*P* < 0.01) exhibited a negative correlation with MNC, whereas the predicted functional abundance (*P* = .667) and number of nodes (*P* = .433) and edges (*P* = .752) showed no significant correlation with MNC.

**Figure 6:**
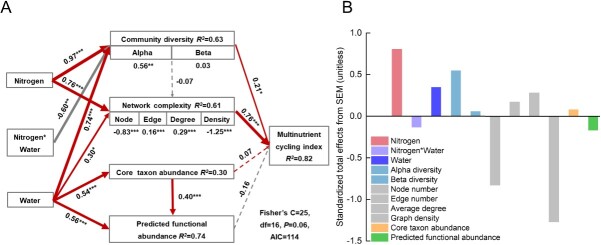
Direct and indirect pathways through which nitrogen–water supply and interaction affected rhizospheric bacterial community and MNC. (A) PiecewiseSEM of community diversity (alpha diversity and beta diversity), core taxon abundance, network complexity (node number, edge number, average degree, and graph density), and MNC as affected by nitrogen and water supply. In Figure A, red and gray lines represent positive and negative relationships, respectively. Solid and dashed lines indicate statistically significant and non-significant relationships, respectively. *P < 0.05, **P < 0.01, ***P < 0.001. (B) Standardized total effects from PiecewiseSEM.

We constructed an *a priori* model that simultaneously considered the effects of nitrogen–water supply and interaction, alpha diversity, beta diversity, core taxon abundance and predicted functional abundance, node number, edge number, average degree, and graph density on MNC (Supplementary Fig. S4). Piecewise structural equation modeling (piecewiseSEM) was employed to elucidate the direct and indirect pathways through which nitrogen–water regulation affected the rhizospheric bacterial community and MNC (Fig. 6). Nitrogen–water supply and interaction together explained 82%, 63%, 61%, 30%, and 74% of the variation in MNC, community diversity, network complexity, core taxon abundance, and predicted functional abundance, respectively (Fig. 6a). Nitrogen and water supply exerted notable positive influences on community diversity and network complexity, while nitrogen–water interaction demonstrated a significant negative impact on community diversity (*P* < 0.05). The standardized total effect analysis revealed that nitrogen was the main factor affecting MNC, and alpha diversity contributed more to MNC than beta diversity (Fig. 6b). Water supply had the strongest effects on predicted functional abundance and core taxon abundance (*P* < 0.001). Compared to predicted functional abundance and core taxon abundance, community diversity and network complexity were found to play crucial roles in regulating responses of MNC to changes in nitrogen and water supply.

## Discussion

As nitrogen and water supply are two major factors that influence microbial communities, changes in nitrogen and water influence soil microbial diversity and function [24, 25]. Our results showed that drought conditions resulted in a reduction in microbiota richness and Shannon index compared to waterlogged conditions (Fig. 1a, b). This is in line with prior findings that waterlogging has positive effects on soil microbial diversity, whereas drought can lead to physiological stress and a decrease in community richness [26, 27]. Increased water supply decreases extracellular enzyme activity and intensifies competition for resources [28], while lower water supply indirectly alters community composition and soil properties, thereby diminishing alpha diversity [29]. The bacterial microbiota formed three distinct clusters (drought, normal, waterlogged) based on the water supply level, indicating a clear spatial ecological niche (Fig. 1c). Drought appeared to inhibit microbial activity in the most active layers of the soil, which may limit nutrient and carbon cycling and lead to temporal differences between nutrient supply and plant demand [30]. In general, anoxic environments under waterlogged stress inhibit microbial activities, such as a decrease in the number of microorganisms involved in nitrification reactions [31]. However, nitrification was not inhibited under the waterlogged treatment (Fig. 2b). This is because the root oxygen supply was able to satisfy some of the microbial respiration during low-oxygen conditions [32]. The activities of microbial extracellular enzymes for carbon, nitrogen, and phosphorus acquisition are enhanced under lower oxygen concentrations, thereby facilitating the regulation of plant nutrient uptake by rhizosphere microorganisms [33]. We found the bacterial richness and abundance in waterlogged conditions with nitrogen fertilization to be slightly lower compared to those in waterlogged conditions with nitrogen starvation. This is due to the inhibitory effect of increased nitrogen availability on soil microbial respiration rates under waterlogged conditions [34]. There was a significant relationship between microbial respiration efficiency and microbial richness and abundance [35]. This suggests that inhibition of microbial respiration efficiency may reduce microbial richness and abundance. Therefore, N fertilization under waterlogged conditions may have a potential inhibitory effect on the bacterial community.

The observed neutral response of alpha diversity to nitrogen fertilization is inconsistent with prior findings suggesting a negative effect of nitrogen fertilization on microbial diversity [36, 37]. There are three reasons for this phenomenon. First, nitrogen inputs may alleviate nitrogen limitations on plant and microbial growth, but intense competition between plants and microbes for additional limiting resources (i.e. water and carbon) may counteract the positive impacts of increased nitrogen efficacy on microbial diversity [24, 38]. Second, in the short term, high nitrogen inputs to soils can induce soil acidification and cause ammonium toxicity to soil microorganisms [37, 39]. The microbial communities, however, may be on the verge of reaching a critical point where they can adapt to increased nitrogen toxicity and thus prevent a decline in diversity [24]. Third, the application of nitrogen induces changes in species replacement and abundance but may not result in species loss or gain, ultimately exerting a neutral impact on microbial diversity [40]. In line with previous findings [41], we observed that water exerted a more significant influence on rhizospheric microbial diversity than nitrogen (Fig. 1a–c); either due to changes in water supply having a pronounced impact on root morphology and metabolism [42] or the limited responsiveness of bacteria to nitrogen supply compared to other environmental factors [43, 44]. Notably, we observed a remarkable effect of water supply on quantitative bacterial abundance, whereas nitrogen supply had no significant effect (Fig. 1d). This is partly because we applied urea, a rapidly acting nitrogen source, at a low rate, resulting in limited effects on the bacterial populations [45].

Associations in co-occurrence networks imply interactions among microorganisms or a common ecological niche [46, 47]. In this study, the number of edges, average degree, and graph density of the microbial network were reduced under waterlogged conditions when compared to those under drought (Fig. 3h, i, j), suggesting that the tomato rhizosphere under moderate drought may promote bacterial interactions or the formation of additional shared ecological niches, which affect plant performance. Interestingly, we did not observe an increase in network complexity in response to increased nitrogen fertilization (Fig. 3g–j), in contrast to prior observations that increased resource availability improves network complexity [48]. This can be explained from several perspectives. First, water supply is more effective than nitrogen supply in expanding niche breadth, thus mitigating competing interactions that affect network complexity [49]. Second, nitrogen fertilization can suppress the growth of certain functional microorganisms, such as nitrogen-fixing and denitrifying bacteria, thereby simplifying the network among microbial communities [24]. Third, increased nitrogen availability may shift species interactions from more competition to reciprocal symbiosis, provided there is sufficient ecological niche space [36, 50, 51]. Overall, rhizospheric bacterial community assemblages were more sensitive to water than to nitrogen.

The abundance of rhizospheric core bacterial taxa is crucial for the process of nutrient cycling [52]. The current study underscored the association between the abundance of core taxon and plant multinutrient traits (Fig. 4). Some of these bacteria (i.e. *Bacillus*, *Streptomyces*, *Mesorhizobium*, *Sphingomonas*, and *Rhizobium*) promote plant growth, solubilize nutrients such as P and Ca, and produce various phytohormones and siderophores [53]. While it is true that a correlation does not necessarily imply causation, our results revealed notable associations between the abundances of several core genera and specific plant nutrients (Fig. 4). For instance, *Candidatus Koribacter* is positively correlated with Fe concentration because it is an avid rhizosphere colonizer and can produce siderophores [54]. We also observed strong correlations between *Streptomyces* and Zn concentration owing to the important role of *Streptomyces* in predicting changes in Zn concentration [16]. The core taxa inhabiting the rhizosphere can frequently serve as potential candidate members for the design of microbial communities with biofertilizers [55]. In addition, core microbial taxon can be considered an important factor in improving agricultural sustainability and crop productivity [22]. In summary, the members of the core bacterial genera in this study provide valuable insights for the selection of candidate taxa in future agricultural microbiome engineering solutions.

Increased alpha and beta diversity were positively associated with the MNC (Fig. 5), validating the biodiversity–ecosystem function hypothesis that heightened microbial diversity improves ecosystem functioning [56]. Moreover, increased soil microbial diversity has been previously associated with better nutrient availability [57]. A positive relation between microbial diversity and multifunctionality has been identified in high-intransitive communities characterized by weak competitive interactions [58]. Our results provide strong evidence that changes in microbial diversity directly impact nutrient availability. Furthermore, in line with previous observations [19], our findings indicate a positive correlation between core taxon abundance and MNC, suggesting that core taxa drive changes in multiple nutrient concentrations, underscoring their diverse functions in agroecosystems. Complex microbial networks are widely acknowledged as a critical element in sustaining ecosystem functions [59, 60]; however, there is a lack of data to support this. A few studies have quantified the relationships between network topological features and plant nutrient concentrations [61, 62]. In this study, the number of network edges, average degree, and graph density were negatively correlated with P and Mn concentrations (Supplementary Fig. S5), which contrasts with previously established positive relationships between network topological features and nutrient uptake. This is due to the fact that under abiotic stress conditions, bacteria with similar environmental and resource preferences form weakened interactions in the microbial network, which in turn reduces resource utilization and information transfer [63]. In summary, plant multinutrient traits are closely dependent on rhizospheric bacterial community assemblages.

Abiotic factors can indirectly affect changes in nutrient concentrations [64]. For example, water supply indirectly affects nutrient concentrations by changing community composition [65]. Microbial diversity indices (i.e. richness and Shannon) are important predictors of multinutrient traits and were positively associated with MNC [16, 66]. Compared to predicted functional abundance and core taxon abundance, community diversity and network complexity were found to play crucial roles in regulating the responses of multinutrient traits to changes in nitrogen and water supply (Fig. 6), which is consistent with previous research [67, 68]. The increase in bacterial taxa leads to a corresponding increase in both positive or negative biotic interactions such as facilitation, niche complementation, and competition, all of which regulate the efficiency of resource use [69]. Therefore, a higher microbial diversity can ensure that terrestrial ecosystems perform their functions better [22]. Furthermore, greater network complexity and associations supported by multiple microbes play a key role in regulating ecosystem multifunctionality [67]. The interaction between microbial communities is also considered an important factor in improving multifunctionality [70], possibly due to the metabolic division of labor among microorganisms, resulting in the complementarity among those with unique physiological properties [71]. This was confirmed by piecewiseSEM, in which community diversity had a significant positive effect on MNC. Furthermore, we observed a direct positive effect of network complexity on MNC (Fig. 6). Network complexity has often been shown to help ensure ecosystem functions, such as nutrient cycling and resisting environmental stress [70, 72]. One possible explanation is that more intricate community associations offer a greater advantage in resource utilization and information transfer to facilitate a diverse array of functions [49]. Plants can recruit specific microbes to alleviate negative effects of environmental stress, according to the ‘cry-for-help’ hypothesis [73]. For instance, under abiotic stress factors, microbiota recruitment by the plant can significantly impact the composition and interactions within the surrounding microbial community, as well as the multifunctionality of the ecosystem [74–76]. This is consistent with our finding that nitrogen and water supply-induced changes in microbiota interactions affect the MNC index. Indeed, community diversity and network complexity form a critical link between abiotic factors (nitrogen and water supply) and MNC, and their positive impacts on MNC imply that they play a role in strengthening nutrient cycling. In conclusion**,** rhizospheric bacterial community diversity, quantitative abundance, predicted functional abundance, and network topological features were more sensitive to water supply than to nitrogen supply. In addition to community diversity, network complexity contributed to multinutrient absorption by host plants under different levels of nitrogen and water supply. Our findings highlight the contributions of rhizospheric bacterial communities in maintaining aboveground multinutrient utilization. These findings enhance our comprehension of soil microbial regulation of plant nutrient traits and provide more explicit directions for the development of multifunctional agro-ecosystems.

## Materials and methods

### Experimental design and management

The greenhouse experiments were performed at the Shenzhen campus of Sun Yat-sen University (Shenzhen, China, latitude 22°52’N, longitude 114°38′E) in 2022. The soil was a laterite soil, which contains 5.87 pH, 0.54 g kg^−1^ total N, 100.61 mg kg^−1^ available P, and 128.51 mg kg^−1^ available K.

Using a randomized block design with nitrogen and water treatments, we combined two nitrogen treatments, i.e. nitrogen starvation (no nitrogen fertilization) and nitrogen fertilization (0.3 g per pot), with three irrigation conditions, i.e. drought (Dro.), normal (Nor.), and waterlogged (Wat.). Therefore, a total of six treatments were incorporated, each with six replicates. In this experiment, seedlings of the cultivar M82, with similar sizes, were transplanted into pots filled with 300 g of dry soil, with one seedling being planted in each pot. There was one plant in each replicate. Nitrogen fertilizer (0.3 g per pot), phosphorus fertilizer (0.65 g per pot), and potassium fertilizer (0.09 g per pot) were applied as basal fertilizers before transplantation [77]. No additional fertilizer was applied during the experiment. Each plant receives 50 ml of water every 2 days to support normal growth prior to water treatment. Two weeks after transplantation, all pots assigned to nitrogen starvation or nitrogen fertilization treatments were randomly divided into drought, normal water, and waterlogged groups. For the normal irrigation treatment, the previous watering regime was maintained, whereas for drought treatment, 25 ml of water was added to each pot every 2 days to simulate moderate water stress (resulting in a soil water content of 45%–55% compared to that of normal water) [78]. The drought stress was maintained for 2 weeks until mild leaf wilting occurred. Similarly, waterlogged stress lasted for 2 weeks, and water was supplied until it reached the pot surface level and was maintained consistently. The growth conditions of the plants remained consistent throughout the same treatment.

### Sample collection and processing

The samples for all treatments were collected 30 days after transplantation, at which point the drought-treated plants showed mild leaf wilting. One plant under the drought plus nitrogen fertilizer treatment had wilted by the end of the experiment, leaving only five samples available for in-depth analysis. The other five treatments all contained six samples. The aboveground parts of the plants were dried at 105°C for 30 min, followed by further drying at 70°C to reach a constant weight. Roots were collected from each pot by cautiously pulling them out of the pot, shaking the roots vigorously to remove loose soil. Roots were cut and transferred to 15-ml centrifuge tubes containing 10 ml of phosphate-buffered saline buffer [78, 79]. The centrifuge tubes were vortexed for 15 s, then the root tissue was gently removed and centrifuged at 4500 × *g* for 15 min. The rhizosphere soil per pot was collected for DNA extraction. A portion of the dried plant tissue was digested with H_2_SO_4_-H_2_O_2_ for the determination of N, P, and K. The remaining parts were digested with HNO_3_-H_2_O_2_ for the determination of Ca, Mg, Zn, Mn, Fe, and Cu. Total plant N was determined using a rapid azotometer, and total plant K was determined using flame photometry. Total plant P was determined using molybdenum blue spectrophotometry (λ = 880 nm).

### Absolute quantitative sequencing

A total of 35 rhizosphere soil samples representing the six treatments were selected for absolute quantitative sequencing. Sequencing was outsourced to Genesky Biotechnology Inc. (Shanghai, China). Briefly, total genomic DNA was extracted using the FastDNA® SPIN Kit for Soil and a FastPrep® instrument (MP Biomedicals, Santa Ana, CA, USA). 16S amplicon library construction and sequencing was performed by adding a certain amount of spike-in standards synthetic sequences to the sample DNA [80, 81]. The raw sequence data were processed separately for each library using the QIIME2 pipeline. Quality filtering, noise reduction, splicing, and de-chimerization of data were performed using the DADA2 method [82], and the quality-filtered reads were assembled into error-corrected amplicon sequence variants (ASVs). A standard curve was plotted based on the number of 16S amplicon reads and their absolute copy numbers of spike-in standards, and the absolute copy numbers of 16S rRNA genes of the species in the samples that were within the range of the standard curve were calculated [83]. Raw read data were submitted to the NCBI Sequence Read Archive (SRA) under BioProject PRJNA1042797.

### Functional characteristics and core taxon of bacterial communities

FAPROTAX was used to predict functional pathways of the microbiota under different treatments, using default settings [84]. The genus that was 100% present in all samples from each treatment was designated as the core taxon [19].

### Co-occurrence pattern construction

To minimize the occurrence of rare ASVs, only ASVs present in >80% of the samples were preserved. Co-associations were established through the construction of Spearman correlation matrices using R package “WGCNA” [85]. Robust correlations with Spearman correlation coefficients of >.7 and false discovery rate-corrected *P*-values of <0.01 were identified to construct networks in which each node represents an ASV and each edge signifies a strong and significant correlation between two nodes [19, 72]. The key topological features of the network (i.e. node number, edge number, average degree, and graph density) for each sample were obtained using the ‘igraph’ package [86]. The number of nodes indicates the number of species in a microbial network. The number of edges represents the number of connections between each pair of species. The average degree indicates the average number of connections per node in the network. The graph density represents the intensity of connections among nodes [87, 88]. The network was visualized using the Gephi software.

### Plant MNC index determination

The plant MNC index was conducted based on multiple aboveground nutrient contents, similar to the widely used multifunctionality index [16, 22]. Plant nutrients, such as N, P, K, Ca, Fe, Mg, Zn, Cu, and Mn, provide some of the fundamental supporting and regulating ecosystem services essential for crop growth [19, 89]. To obtain quantitative MNC index values for each nutrient element, we standardized the nine nutrient traits using Z-Score transformation. The MNC index was calculated as the average of the standardized scores for each individual nutrient [16, 89] and was used to measure multinutrient traits in host plants. Random forest analysis was employed to assess the importance of the core taxon in driving changes in plant nutrient concentrations [19, 90].

### Statistical analysis

The effects of nitrogen and water and their interactive effects on nutrient concentrations were analyzed using two-way ANOVA. Differences in community composition were tested using PERMANOVA. Linear regression analysis was used to establish the relationships of MNC with community diversity, core taxon abundance, predictive functional abundance, and network topological features. Alpha diversity was determined as the mean of richness and Shannon index values converted from the Z-Score. Beta diversity was calculated based on the first axis of the NMDS plot. PiecewiseSEM was employed to evaluate the direct and indirect associations among nitrogen–water supply and interaction, community diversity, core taxon abundance and predicted functional abundance, network complexity, and MNC [91]. We constructed an *a priori* model that simultaneously considered the effects of nitrogen–water supply and interaction, alpha diversity, beta diversity, core taxon abundance and predicted functional abundance, node number, edge number, average degree, and graph density on MNC. Model path coefficients and associated *P*-values were calculated. Models were corrected stepwise based on pathway significance (*P* < 0.05) and overall model strength (0 ≤ Fisher’s C/df ≤ 2 and 0.05 < *P* ≤ 1) [92].

## Supplementary Material

Web_Material_uhae290

## Data Availability

All the raw sequence data were submitted to the NCBI Sequence Read Archive (SRA) database under accession no. PRJNA1042797.
