# Supplementary material for: Diversity and interactions of rhizobacteria determine multinutrient traits in tomato host plants under nitrogen and water disturbances
Source: Hortic Res. 2024 Oct 15;12(2):uhae290. doi: 10.1093/hr/uhae290 (PMC11789527; doi:10.1093/hr/uhae290)
Supplement: Web_Material_uhae290 [file web_material_uhae290.zip › Supplementary_material.docx]

**Supplementary Material**

**Table S1.** Significance of the bacterial community under different treatments based on PERMANOVA. Numbers in bold indicate significant effects at *P* < 0.05. R-values close to 1 indicate dissimilarity between treatments. NsD: nitrogen starvation plus drought; NsN: nitrogen starvation plus normal watering; NsW: nitrogen starvation plus waterlogged condition; NfD: nitrogen fertilization plus drought; NfN: nitrogen fertilization plus normal watering; NfW: nitrogen fertilization plus waterlogged condition.

| Comparison | PERMANOVA | |
| --- | --- | --- |
|  | *R^2^* | *P* |
| NsD vs. NfD | 0.199 | 0.035 |
| NsN vs. NfN | 0.280 | 0.005 |
| NsW vs. NfW | 0.266 | 0.004 |
| NsD vs. NsN | 0.385 | 0.003 |
| NsD vs. NsW | 0.558 | 0.002 |
| NsN vs. NsW | 0.561 | 0.005 |
| NfD vs. NfN | 0.397 | 0.004 |
| NfD vs. NfW | 0.579 | 0.005 |
| NfN vs. NfW | 0.398 | 0.004 |


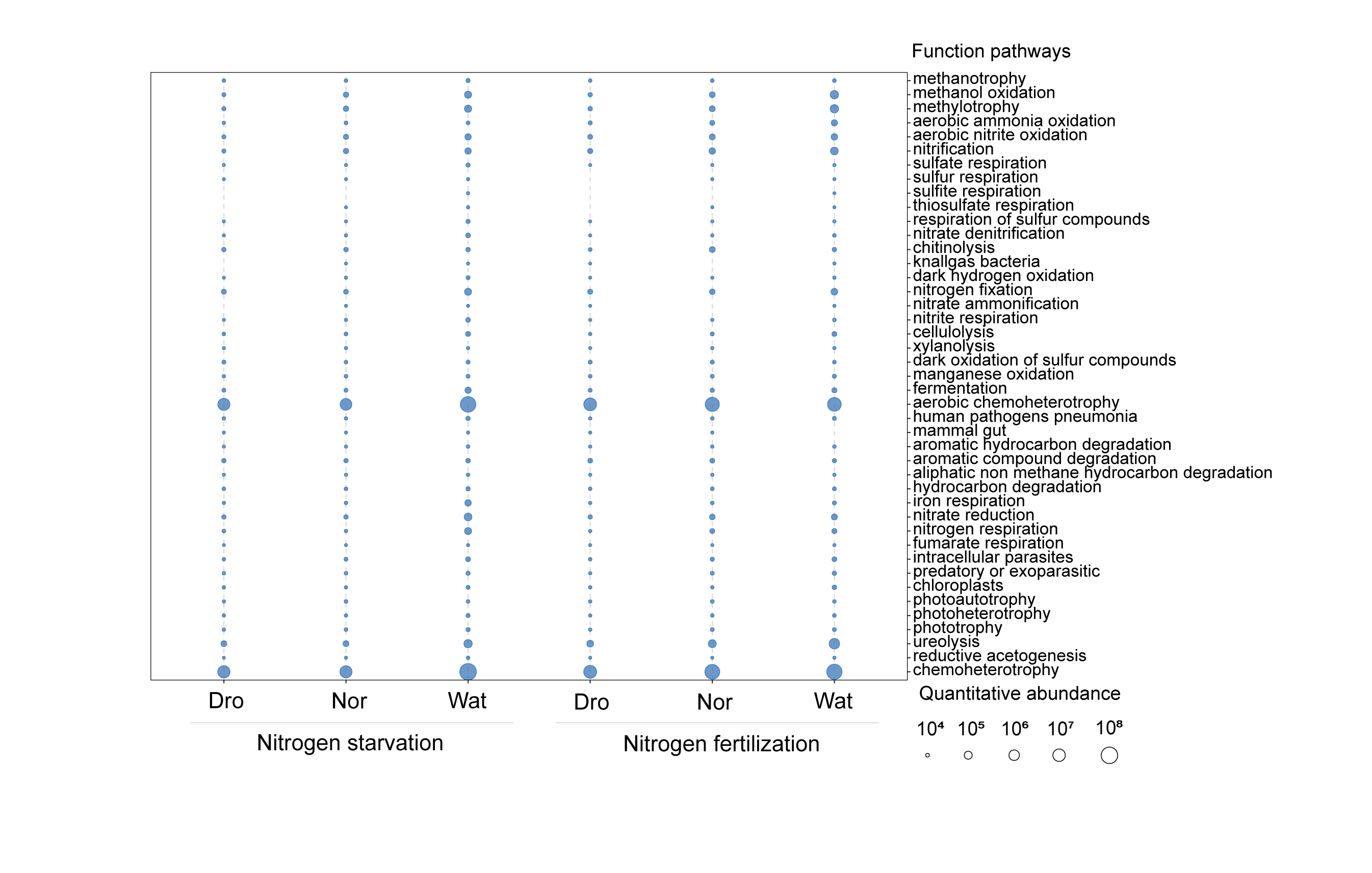


**Figure S1.** Predicted functional pathways of rhizospheric bacterial community. The circle size represents the quantitative abundance of functional pathways.


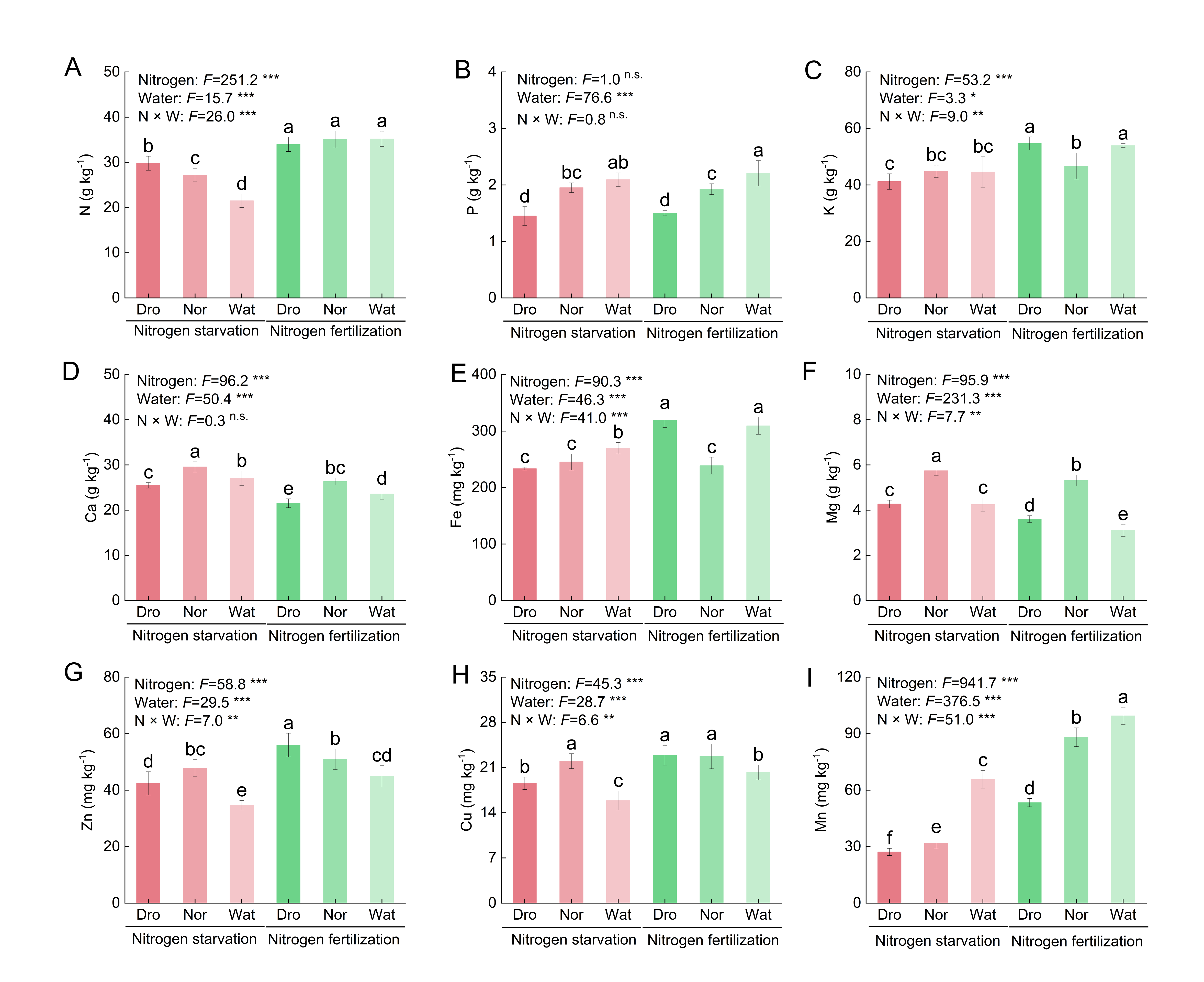


**Figure S2.** Tomato plant nutrient contents under different nitrogen and water supply. Same letters indicate no significant difference at *P* < 0.05. * *P* < 0.05, ** *P* < 0.01, *** *P* < 0.001. ns, not significant.


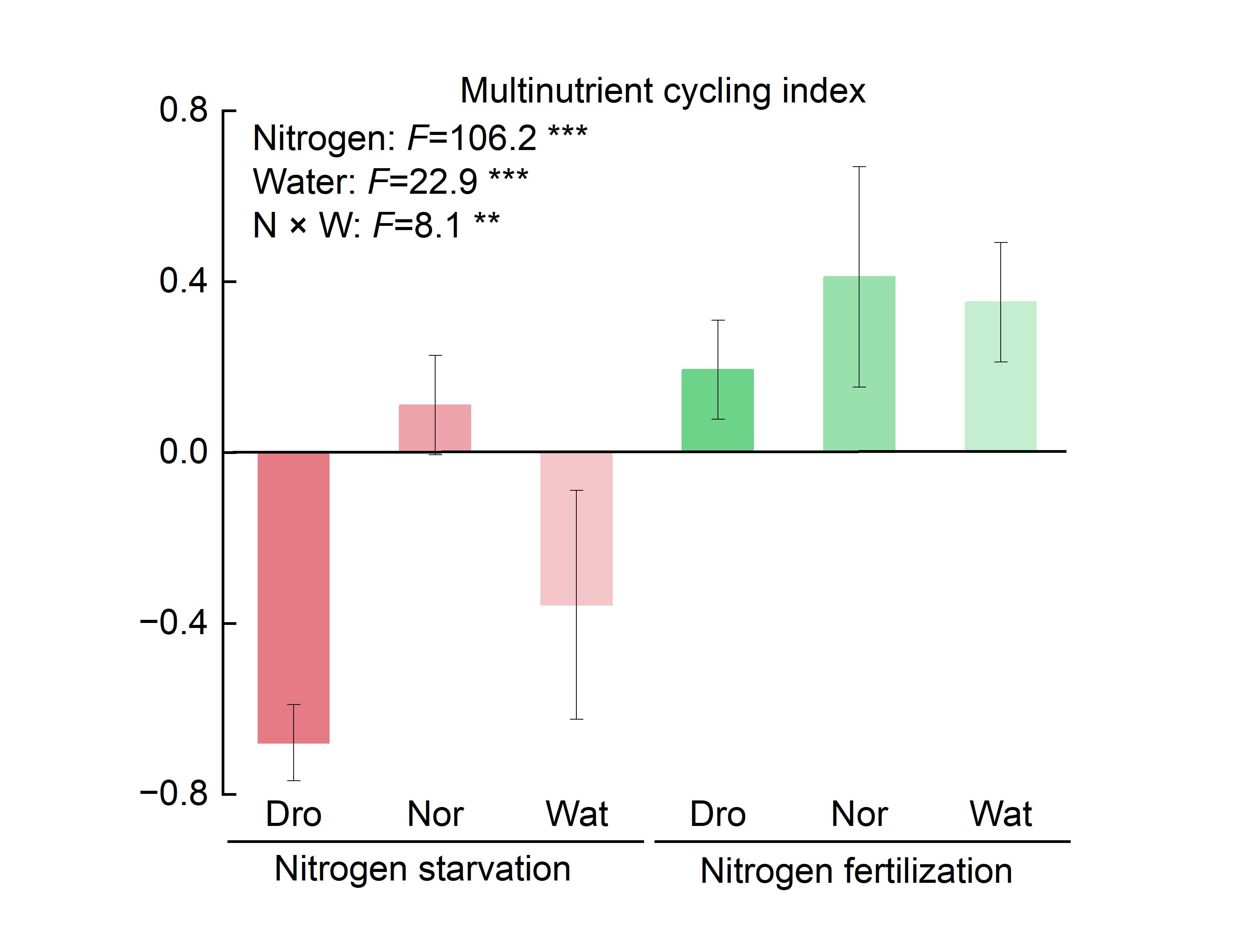


**Figure S3.** Multinutrient cycling index (Z-Score) under different nitrogen and water supply.


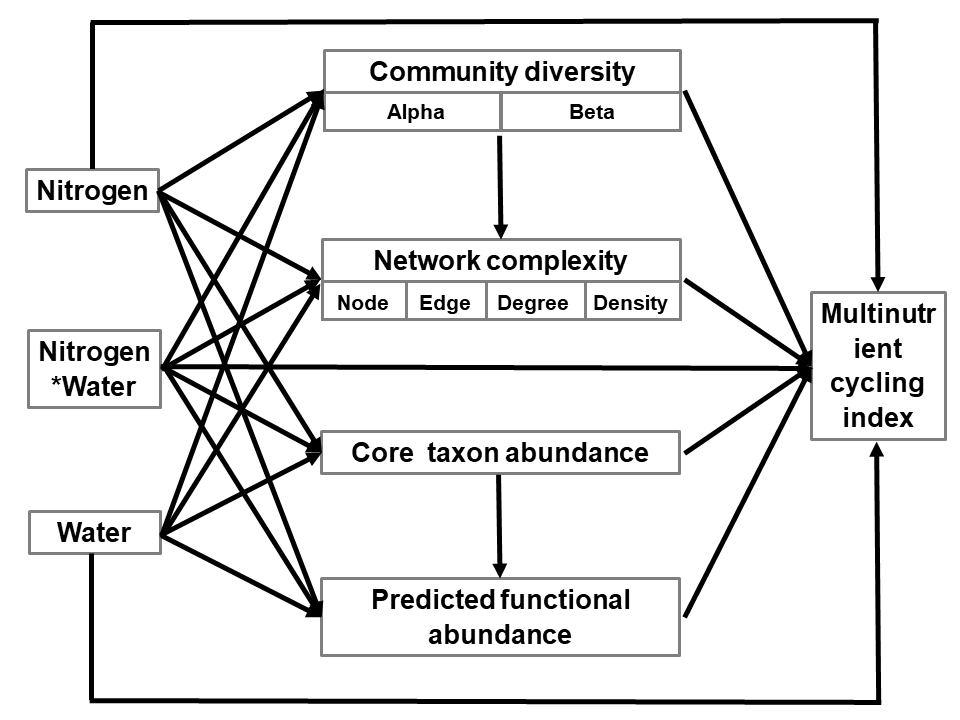


**Figure S4.** A priori generic structural equation model (SEM) used.


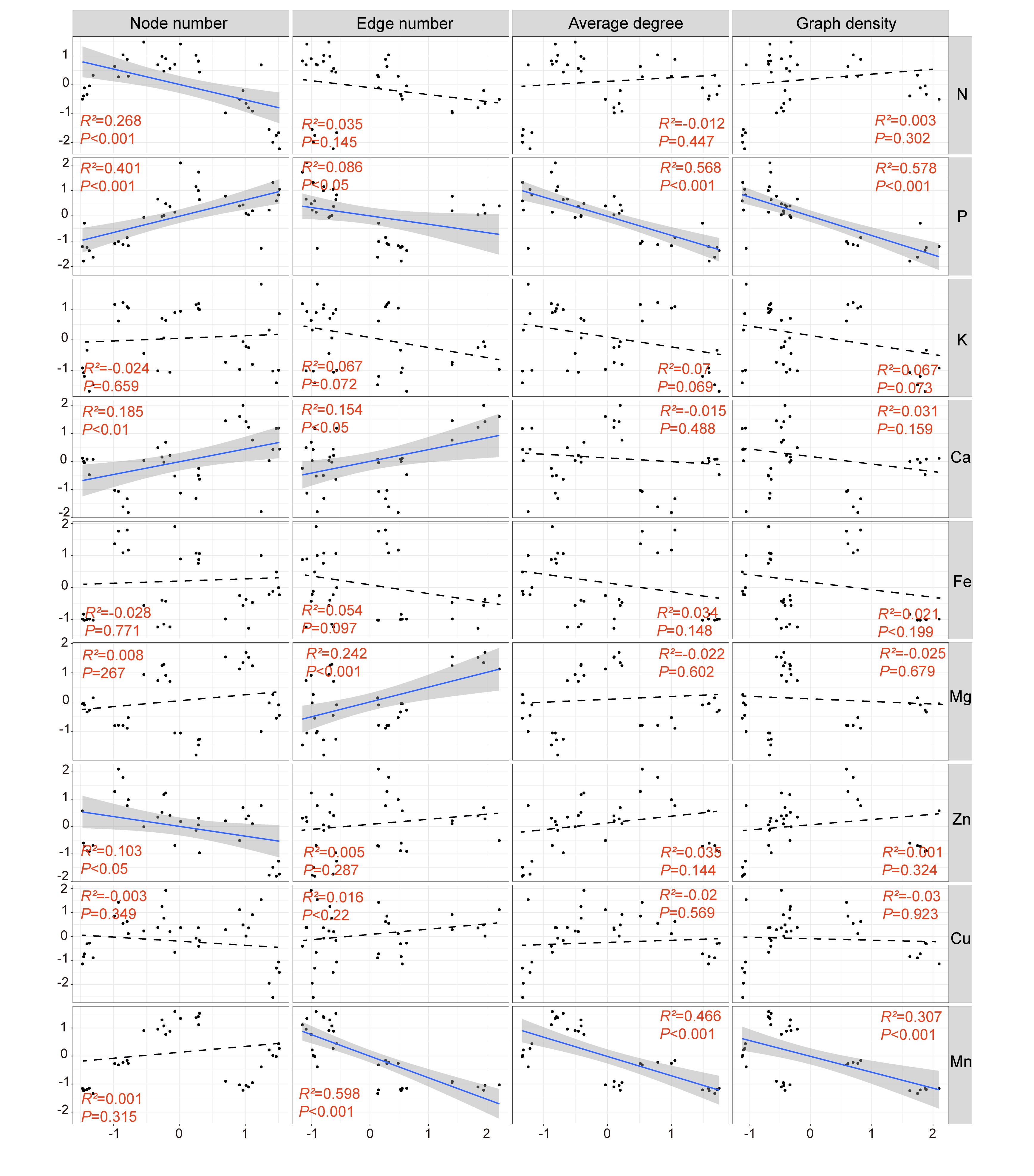


**Figure S5.** Relationships between plant nutrients and the number of nodes and edges, average degree, and graph density. Gray-shaded regions depict 95% confidence intervals. Blue lines denote significant Pearson correlations (*P* < 0.05). Dashed lines denote non-significant Pearson correlations (*P* > 0.05).
