# Supplementary figures and images for: Diversity and interactions of rhizobacteria determine multinutrient traits in tomato host plants under nitrogen and water disturbances
Source: Hortic Res. 2024 Oct 15;12(2):uhae290. doi: 10.1093/hr/uhae290 (PMC11789527; doi:10.1093/hr/uhae290)

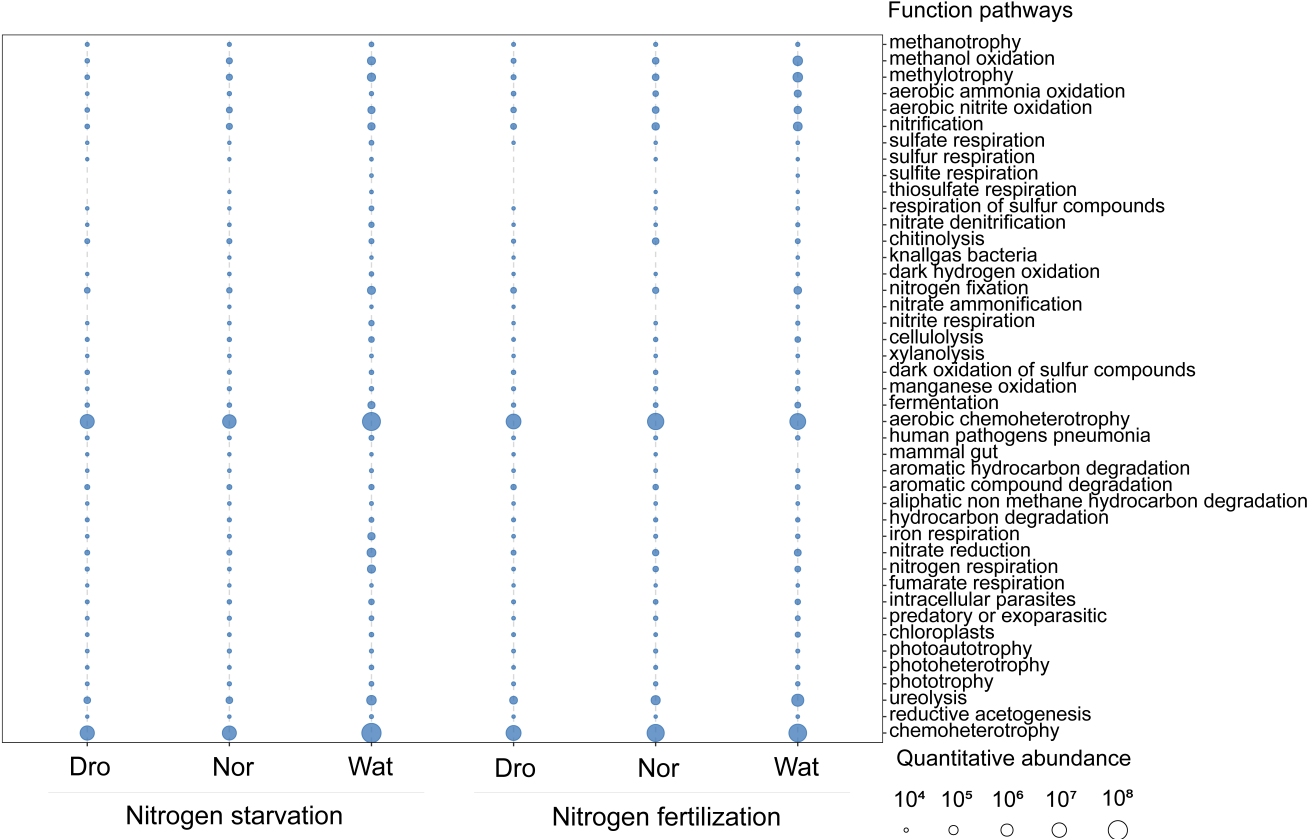

Supplement: Web_Material_uhae290 [file web_material_uhae290.zip › Fig S1.pdf]

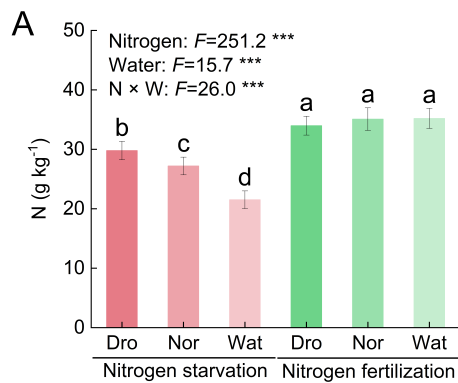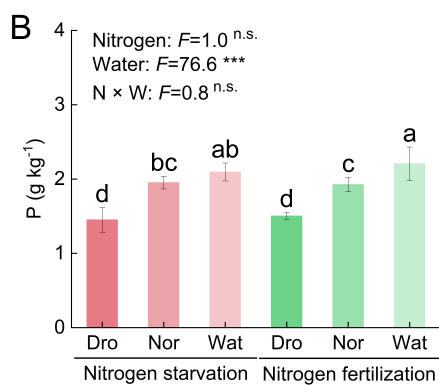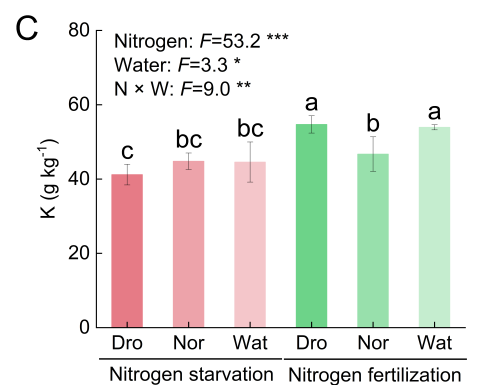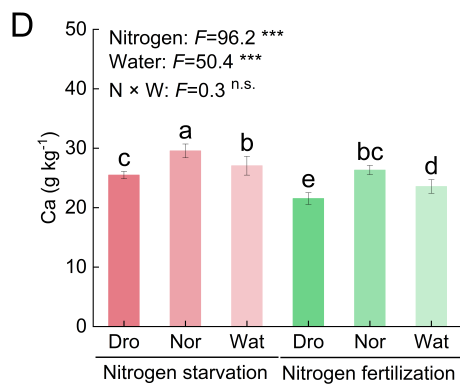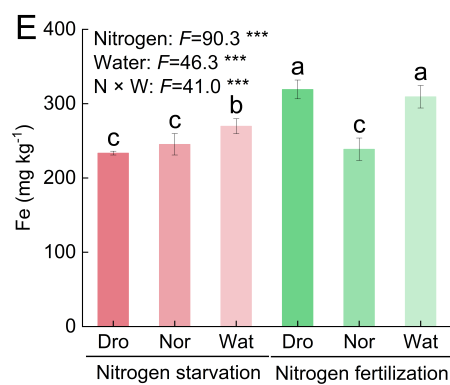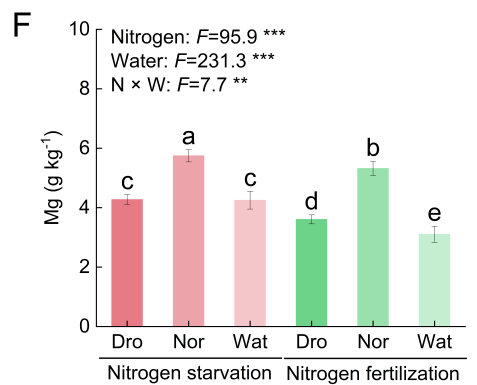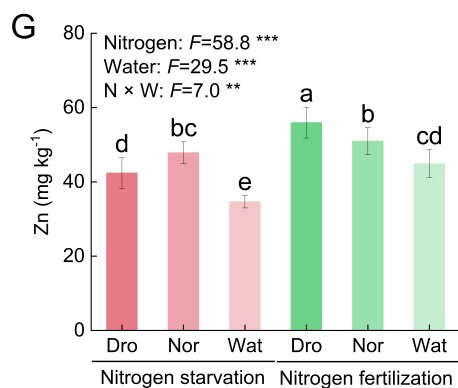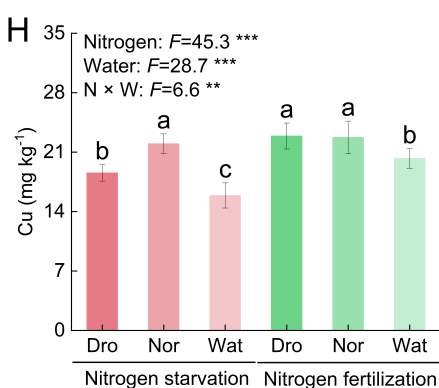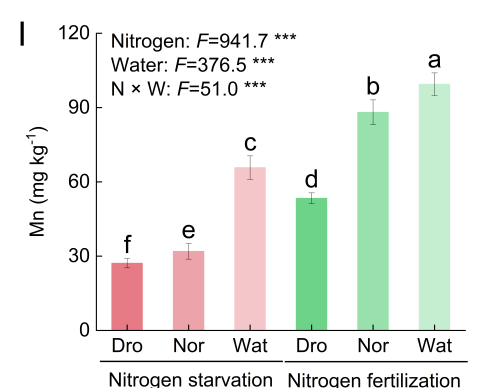

Supplement: Web_Material_uhae290 [file web_material_uhae290.zip › Fig S2.pdf]

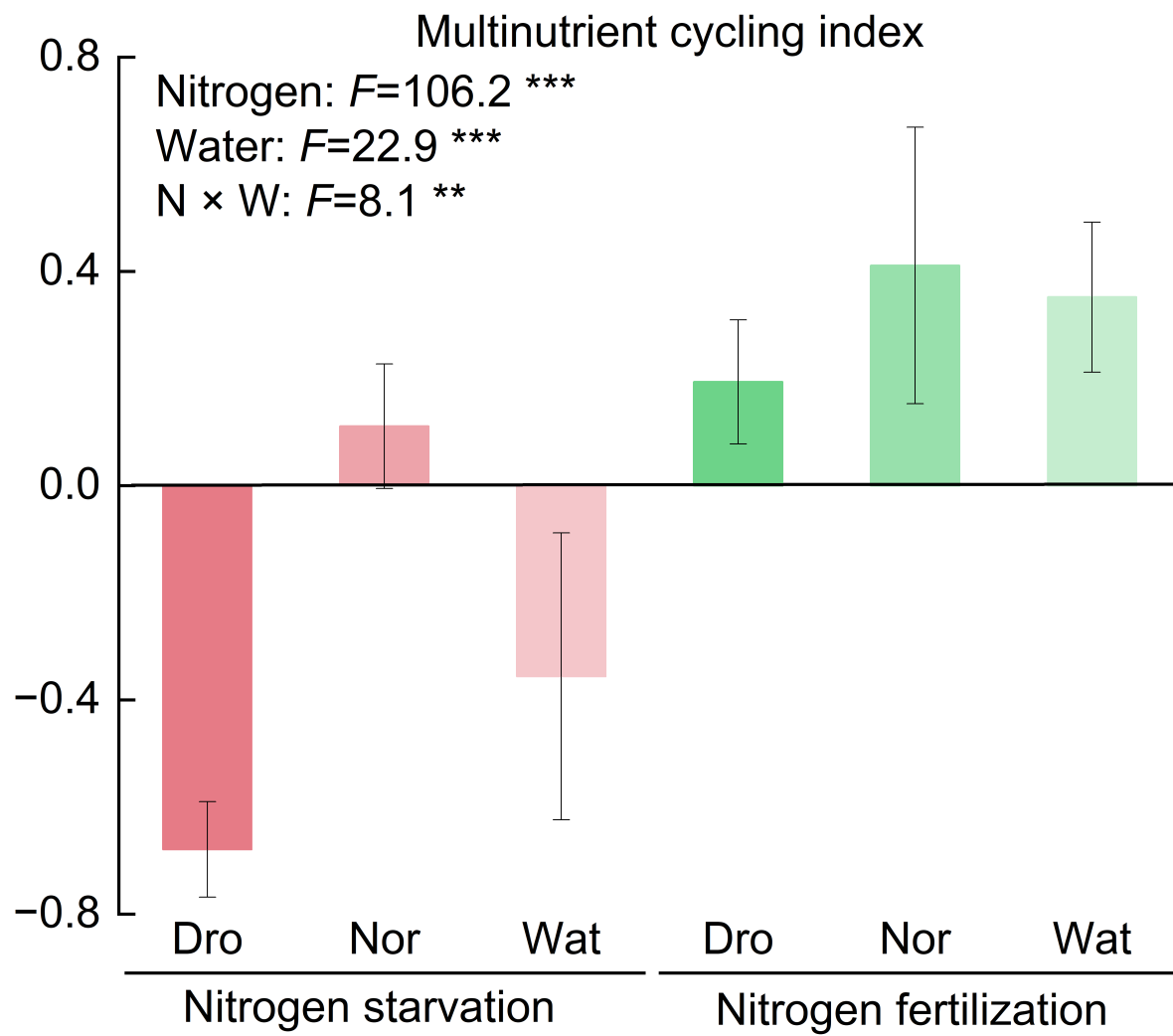

Supplement: Web_Material_uhae290 [file web_material_uhae290.zip › Fig S3.pdf]

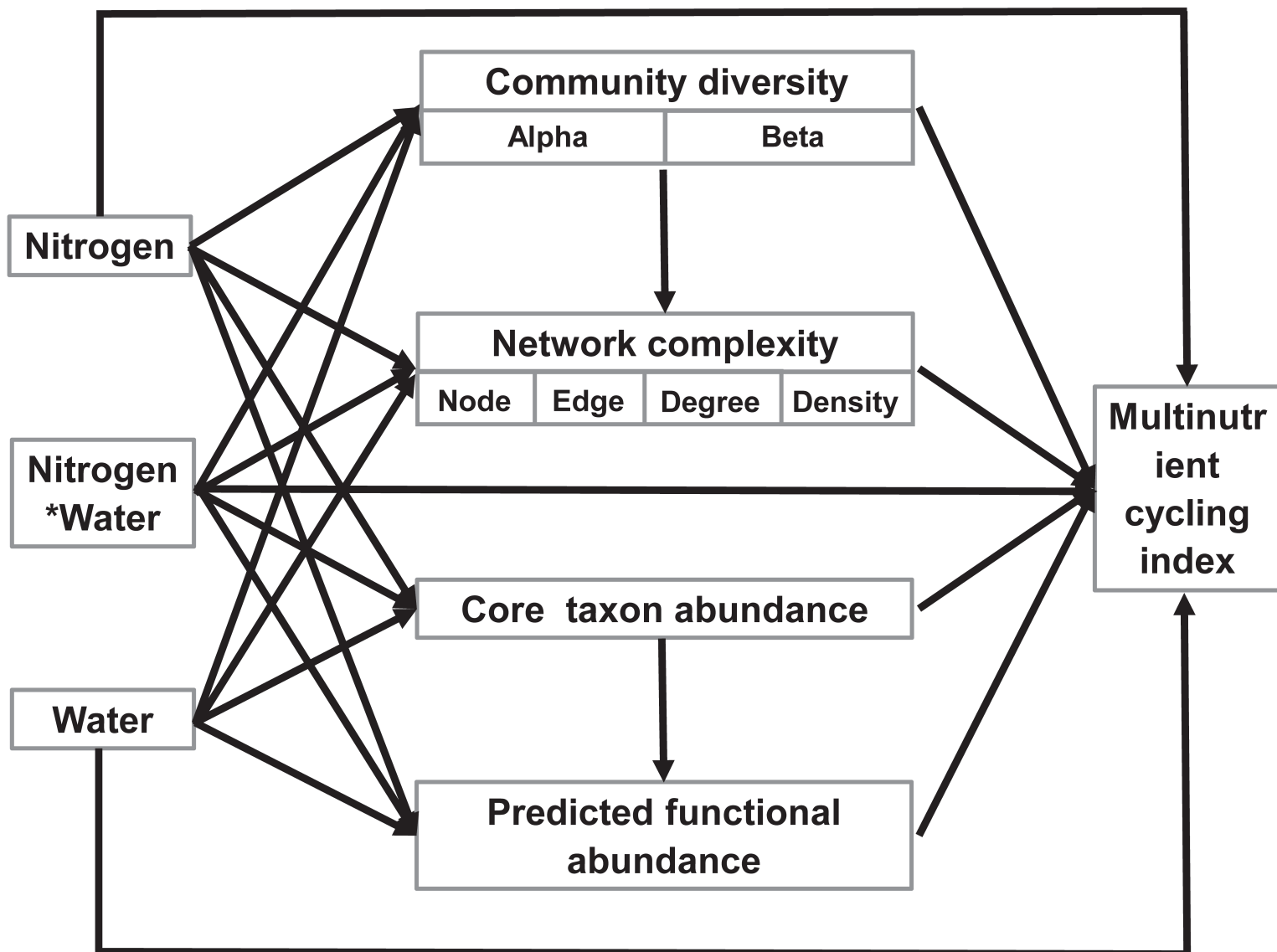

Supplement: Web_Material_uhae290 [file web_material_uhae290.zip › Fig S4.pdf]

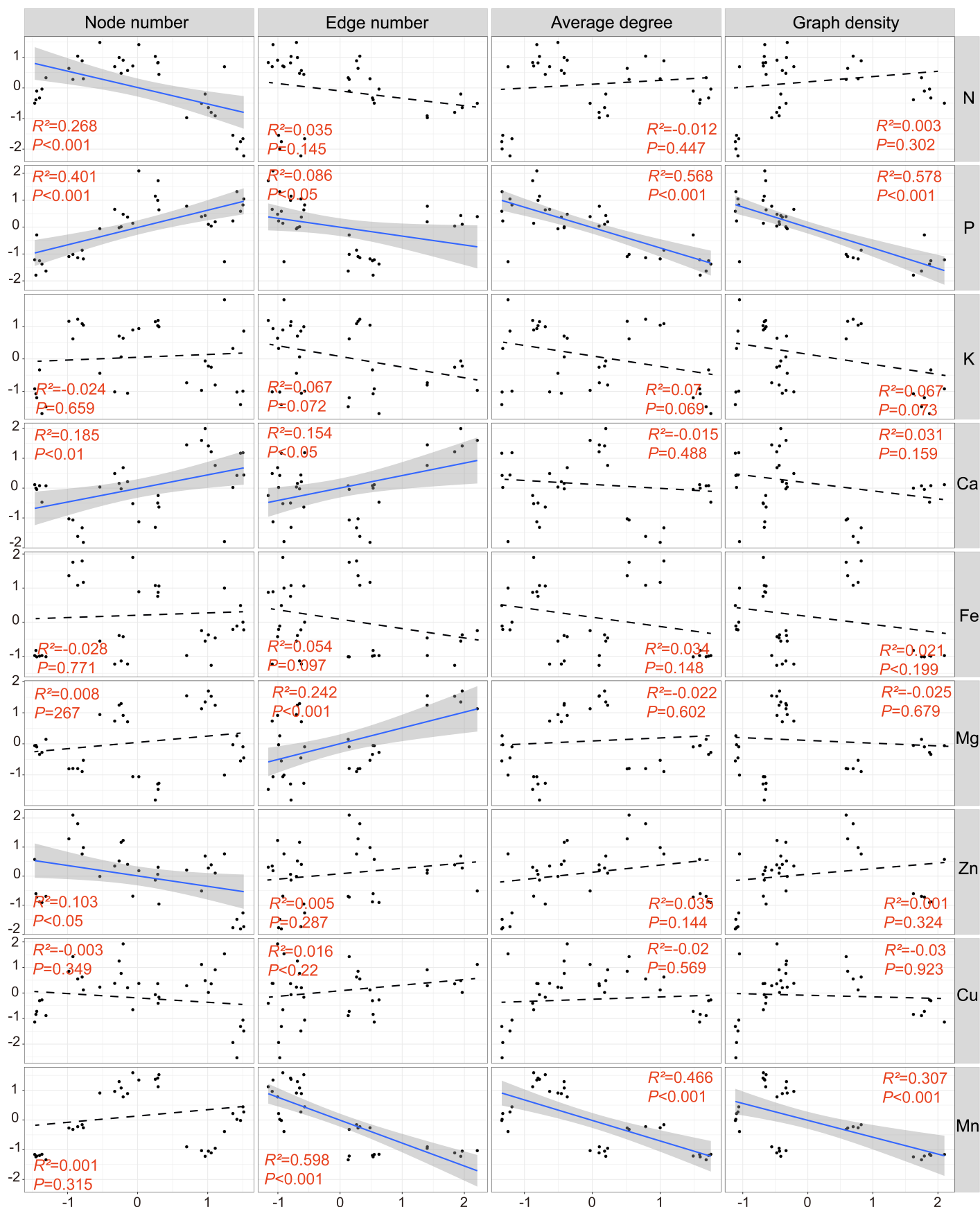

Supplement: Web_Material_uhae290 [file web_material_uhae290.zip › Fig S5.pdf]
